# Improved adherence adjustment in the Coronary Drug Project

**DOI:** 10.1186/s13063-018-2519-5

**Published:** 2018-03-05

**Authors:** Eleanor J. Murray, Miguel A. Hernán

**Affiliations:** 1 Department of Epidemiology, Harvard T.H. Chan School of Public Health, 677 Huntington Ave, Suite 911, Boston, MA 02115 USA; 2 Department of Biostatistics, Harvard T.H. Chan School of Public Health, Boston, MA USA; 3 Harvard-MIT Division of Health Sciences and Technology, Boston, MA USA

**Keywords:** Per-protocol effect, Intention-to-treat effect, Inverse-probability weighting, Coronary Drug Project, Adherence

## Abstract

**Background:**

The survival difference between adherers and non-adherers to placebo in the Coronary Drug Project has been used to support the thesis that adherence adjustment in randomized trials is not generally possible and, therefore, that only intention-to-treat analyses should be trusted. We previously demonstrated that adherence adjustment can be validly conducted in the Coronary Drug Project using a simplistic approach. Here, we re-analyze the data using an approach that takes full advantage of recent methodological developments.

**Methods:**

We used inverse-probability weighted hazards models to estimate the 5-year survival and mortality risk when individuals in the placebo arm of the Coronary Drug Project adhere to at least 80% of the drug continuously or never during the 5-year follow-up period.

**Results:**

Adjustment for post-randomization covariates resulted in 5-year mortality risk difference estimates ranging from − 0.7 (95% confidence intervals (CI), − 12.2, 10.7) to 4.5 (95% CI, − 6.3, 15.3) percentage points.

**Conclusions:**

Our analysis confirms that appropriate adjustment for post-randomization predictors of adherence largely removes the association between adherence to placebo and mortality originally described in this trial.

**Trial registration:**

ClinicalTrials.gov, Identifier: NCT00000482. Registered retrospectively on 27 October 1999.

**Electronic supplementary material:**

The online version of this article (10.1186/s13063-018-2519-5) contains supplementary material, which is available to authorized users.

## Background

In 1980, an analysis of the Coronary Drug Project (CDP) randomized trial found a greater survival in individuals who adhered to placebo than in those who did not adhere to placebo [1]. Statistical adjustment for multiple prognostic factors could not remove these survival differences. This finding was used to support the thesis that adherence adjustment in randomized trials is not generally possible and, therefore, that only intention-to-treat analyses should be trusted.

In 2016, we reported a re-analysis of the CDP placebo arm that incorporated statistical advances not available in 1980. Our re-analysis showed that most of the survival differences between placebo adherers and non-adherers were actually removed after adjustment for pre- and post-randomization prognostic factors [2]. Specifically, we modified the definition of adherence at missed visits, updated the method of adjustment for baseline predictors of adherence, and added adjustment for post-randomization predictors of adherence via inverse-probability (IP) weighting.

With these changes, the 5-year survival difference between placebo adherers and non-adherers decreased from 10.9 percentage points (95% confidence interval, 7.5, 14.4) to 2.5 percentage points (95% confidence interval, − 2.1, 7.0).

However, our re-analysis did not take full advantage of the recent methodological advances. In an attempt to conduct a re-analysis as comparable as possible to the original 1980 analysis, we used a cumulative incidence model that did not account for the exact time of death during the 5-year period, and a somewhat unintuitive measure of adherence – a binary indicator for cumulative adherence greater than 80% during the entire 5-year period. Although modeling the data this way was useful to demonstrate that methods developed since 1980 can address previously intractable sources of bias, the analytic approach we used is not the recommended one. Here, we demonstrate a better approach which uses a more natural definition of adherence and a survival analysis approach to incorporate time of death.

### A brief introduction to the Coronary Drug Project

The CDP was a six-arm double blind, placebo-controlled randomized trial of 8341 men with a history of myocardial infarction (MI) enrolled between 1966 and 1969 [3]. Eligibility criteria for participation in the trial included men aged 30–64 years at entry, with an electrocardiogram-documented MI at least 3 months prior to enrollment, a New York Heart Association functional class of I (no limitation in physical activity) or II (slight limitations only), no prior surgery for coronary artery disease, no anti-coagulant therapy or lipid-influencing therapy use at entry, and no other chronic medical conditions which could affect trial participation. In addition, potential participants were required to complete a 2-month control period during which all individuals were given placebo and only those who adhered to at least 80% were eligible for randomization. Participants were then randomized within study center and by risk group (low risk if one prior MI with no complications; high risk if more than one MI or complications).

Study medications were prescribed as three pills daily at randomization, and dosage was increased to nine pills daily, based on tolerance, over the following 2 months. Adherence was assessed by study clinicians at each visit based on a visual inspection of pill bottles, and recorded as a six-level categorical variable (≥ 80%, 60–79%, 40–59%, 20–39%, 1–19%, none) which was then adjusted for expected dose at that visit [1, 2].

Three of the five active treatment arms were discontinued early because of adverse events (low- and high-dose equine estrogen, and dextrothyroxine) [4–6], and the other two treatments (clofibrate and niacin) were not found to be effective in reducing mortality [7]. The 5-year cumulative incidence of mortality was 20.9% in the placebo arm, 20.0% in the clofibrate arm, and 21.2% in the niacin arm [7].

## Methods

We restricted our analyses to the 2787 men in the placebo arm. Let *t* be an index for visit, where *t* = 0 is the baseline visit and *t* = 14 is the last visit at year 5, with 4-month intervals between each visit. Let *A*_*t*_ be an indicator for adherence ≥ 80% to the protocol-specified placebo dose between *t* and *t* + 1, *Y*_*t*_ an indicator of death between *t* and *t* + 1, *C*_*t +* 1_ an indicator for loss to follow-up defined as three consecutive missed study visits at *t* + 1, *V* a set of 39 variables measured at the time of randomization, and *L*_*t*_ a set of post-randomization variables measured at each *t*. The baseline covariates *V* were adherence during the run-in period, demographics (age, race), lifestyle characteristics (cigarette smoking, physical activity), medical history (risk group, weight, New York Heart Association class, comorbidities, blood pressure), use of non-study medications, laboratory findings, and electrocardiogram findings. All of these variables (except age, race, weight, risk group) were also post-randomization variables *L*_*t*_ at each visit *t*. When an individual missed a study visit, the most recent covariate and adherence values were carried over from the most recent available data, up to three consecutive missed visits. Participants were censored at the expected date of their third consecutive missed study visit.

The choice of 80% as a cut-point for adherence was based on standard practice, as reflected by the use of a run-in period requiring 80% adherence to placebo among all trial participants. Note that, since adherence was assessed as a categorical variable, with the highest category ≥ 80% of prescribed pills taken, higher thresholds were not possible for the binary adherence indicator.

In our primary analysis, we artificially censored individuals when they reported an adherence level that differed from their baseline adherence level, that is, when *A*_*t*_*≠ A*_0_ [8, 9]. We then fit the IP-weighted pooled logistic model for the discrete-time hazards at each time [10]:

$$ \mathrm{logit}\left(\Pr \left[{Y}_{t+1}=1|\ {A}_k={A}_0\ \mathrm{for}\ 0<k\le t,V,{C}_{t+1}=0,{Y}_t=0\ \right]\right)={\uptheta}_{0,\mathrm{t}}+{\uptheta}_1{A}_0+{\uptheta}_2V+{\theta}_3{A}_0t, $$

where θ_0,t_ is a time-varying intercept modeled as a restricted cubic spline of time (knots at 0, 5, 10, 15 visits), and θ_2_ is a vector parameter. The time-varying stabilized weights [11] were defined as:

$$ {SW}_t={\prod}_{k=0}^{t}\frac{f\left({M}_k,\left.{A}_k\right|{A}_0,\bar{A}_{k-1},V,{C}_k=0\right)}{f\left({M}_k,\left.{A}_k\right|{A}_0,\bar{A}_{k-1},V,{\overline{L}}_k,{C}_k=0\right),} $$

where *M*_*t*_ is an indicator for measurement of adherence at visit *t* (1 if measured, 0 otherwise), and overbars indicate history of the variable. The weight models were fit in the full population before artificial censoring; in a sensitivity analysis, we restricted the fit of the weight models to person-visits with *A*_*k*_*= A*_0_ for 0 < *k* ≤ *t.*

To estimate the denominator of the weights, we fit the model logit(Pr[*M*_*t*_ = 1|${A}_0,{\bar{A}}_{t-1},V,{\overline{L}}_t,{C}_t=0$]) = α_0t_ + α_1_*A*_0_ + α_2_*A*_*t* − 1_ + α_3_*V* + α_4_*L*_*t* − 1_ to all person-visits, and the model logit(Pr[*A*_*t*_ = 0|${A}_0,{\bar{A}}_{t-1},V,{\overline{L}}_t,{C}_t=0$,*M*_*t*_ = 1]) = β_0t_ + β_1_*A*_0_ + β_2_*A*_*t* − 1_ + β_3_*V* + β_4_*L*_*t*_ to the person-visits with measured adherence. When adherence was not measured at a visit (*M*_*t*_ = 0) but the individual was not yet defined to be lost to follow-up (that is, at the first or second consecutive missed visit), adherence was carried forward from the previous visit and the factor in the denominator of the adherence weight was 1 for that visit.

Similar models that did not include the time-varying covariates were fit to estimate the numerators of the weights. The final weight for each individual at each time was the product of the measurement and adherence weights for that individual up to that time point. As in previous studies, we truncated the estimated IP weights at the 99th percentile to avoid undue influence of outliers. The truncated weight estimates had a mean of 1.00 (SD = 0.29) and a range of 0.02 to 2.55.

We used the parameter estimates from the weighted outcome logistic model to estimate the 5-year survival as previously described [2]. We compared the survival for always vs. never at least 80% adherent, that is, *A*_0_ = 0 vs. *A*_0_ = 1.

We conducted a second analysis where, rather than censor individuals who reported a change in adherence, we specified a dose-response function for the effect of adherence on mortality. To do so, we summarized the adherence history *Ā*_*t*_ between baseline and visit *t* by the cumulative average cum(*Ā*_*t*_) = $\frac{1}{t+1}\sum \limits_{k=0}^t{A}_k$ (i.e., the proportion of visits during which an individual was adherent to at least 80% of the placebo dose), and then fit the pooled logistic model

$$ \mathrm{logit}\left(\Pr \left[{Y}_{t+1}=1|{\bar{A}}_t,V,{C}_{t+1}=0,{Y}_t=0\ \right]\right)={\uptheta}_{0,\mathrm{t}}+{\uptheta}_1\mathrm{f}\left[\mathrm{cum}\left({\bar{A}}_t\right)\right]+{\uptheta}_2V, $$

where f[·] is a dose-response function and θ_1_ is a vector parameter.

In separate analyses, we specified more flexible dose-response functions. Specifically, we considered both a quadratic dose-response function θ_1_f[cum(*Ā*_*t*_)] = θ_1,1_cum(*Ā*_*t*_) + θ_1,2_[cum(*Ā*_*t*_)]^2^, and a function that allowed for a separate effect of recent adherence θ_1,0_*A*_*t*_ + θ_1,1_cum(*Ā*_*t* - 1_) + θ_1,2_[cum(*Ā*_*t* - 1_)]^2^. We also considered functions that included product terms with the time parameters.

To compute 95% confidence intervals, we used non-parametric bootstrapping with 500 samples.

SAS 9.4 was used for all analyses and code is provided in the supplementary online materials (see Additional file 1).

## Results

Using our primary hazards models with artificial censoring, the estimated 5-year mortality risk difference between adherers and non-adherers in the placebo arm was 0.01 percentage points (95% confidence interval, -12.2, 13.2) after adjusting for post-randomization covariates (Table 1). Estimates based on dose-response hazards models without artificial censoring gave consistent estimates ranging from less than 1 percentage point to approximately 5 percentage points. Using an oversimplified dose-response model (a linear term only for cumulative adherence) resulted in implausible risk estimates (data not shown). The results were robust to other modeling choices, such as the number or placement of knots for the spline of time. As a sensitivity analysis, we also assessed censoring individuals for loss to follow-up at the second missed visit. Results were similar with an estimated 5-year mortality risk difference of 8.8 percentage points (95% CI, − 1.7, 20.7) in the unadjusted analysis and 0.6 percentage points (95% CI, − 8.1, 10.5) after adjusting for post-randomization covariates. When we restricted the fit of the weight models to person-visits with *A*_*k*_*= A*_0_ for 0 < *k* ≤ *t*, the 5-year mortality risk difference adjusted for post-randomization covariates increased slightly to 3.1 percentage points (95% CI, − 3.1, 9.4).

**Table 1:** Estimated difference in 5-year mortality risk (95% confidence interval) when comparing 0% vs. 100% of follow-up being at least 80% adherent, Coronary Drug Project

Method	Unadjusted	Standardized by baseline covariates	+ IP weighting for post-randomization covariates
Censoring when binary adherence level deviates from baseline	9.6 (– 7.0, 28.9)	1.5 (– 11.0, 16.4)	0.01 (– 12.2, 13.2)
Dose-response hazards models			
Quadratic cumulative adherence	6.7 (− 3.2, 16.5)	2.6 (− 5.8, 11.1)	0.2 (− 8.2, 8.6)
Quadratic cumulative adherence, with time interaction	6.0 (− 5.8, 18.0)	1.6 (− 8.4, 11.6)	− 0.7 (− 12.2, 10.7)
Binary current adherence, plus quadratic cumulative adherence up to previous visit	11.6 (− 1.0, 24.2)	6.4 (− 3.9, 16.8)	4.5 (− 6.3, 15.3)
Binary current adherence, plus quadratic cumulative adherence up to previous visit, with time interaction	10.3 (− 2.3, 22.9)	4.0 (− 6.0, 14.1)	2.4 (− 9.3, 14.1)

## Discussion

Our analysis confirms that the survival differences between adherers and non-adherers to placebo can be largely adjusted away in the Coronary Drug Project. Adjustment for post-randomization predictors of adherence results in estimated 5-year mortality risk differences comparing adherence and non-adherence to placebo that are approximately null regardless of the analytic approach used. Our previous analysis [2] reached a similar conclusion but, by simply estimating the cumulative mortality at the end of follow-up, ignored the timing of events. In contrast, the current analyses use a survival analysis approach that allows estimation of adjusted survival curves for adherers and non-adherers throughout the follow-up.

We used two methods to compare the survival under continuous high-average adherence vs. low adherence (< 80%). The first method was a relatively inefficient censoring procedure that does not require a dose-response model for the effect of cumulative adherence on mortality. The second method was a theoretically more efficient method that requires a dose-response model and, therefore, will result in bias if the dose-response model is misspecified. We considered a variety of dose-response functions, which made different assumptions about the relationship between adherence and mortality over time. When sufficient data are available, more flexible dose-response functions are generally preferable as they impose fewer *a priori* constraints. For example, a function that models separately current adherence and prior cumulative adherence (using, say, linear and quadratic terms as in our analysis) requires fewer assumptions than a function that models total cumulative adherence. However, the added flexibility comes at the price of less precise estimates. Unlike in other examples [12], our estimates were not very sensitive to the choice of dose-response model. Further research into dose-response modeling for adherence-adjusted estimates is warranted.

Obtaining a null estimate when comparing adherers and non-adherers in the placebo arm supports the validity of adherence-adjusted effect estimates in randomized trials. However, this comparison relies on the lack of psychobiological effects of placebo on mortality. For other outcomes, such as pain or symptom severity, this assumption may be less reasonable.

## Conclusion

Adherence-adjusted analyses of randomized controlled trials have been viewed with some skepticism with some authors suggesting that adherence is intractably confounded [1]. However, we have demonstrated that adjustment for post-randomization predictors of adherence can create comparability between adherers and non-adherers when rich data on pre-and post-randomization confounders exist.

## Additional file

Additional file 1: SAS 9.4 code for placebo-arm adherence analysis. A SAS code for all analyses is provided in this file. If you have any questions, comments, or discover an error, please contact Eleanor Murray at emurray@mail.harvard.edu. For the most updated versions of related SAS programs, please visit www.hsph.harvard.edu/causal/. (PDF 316 kb)

## Abbreviations

CDP: Coronary Drug Project
CI: Confidence interval
IP: Inverse-probability

## References

[1] Coronary Drug Project Research Group (1980). Influence of adherence to treatment and response of cholesterol on mortality in the coronary drug project. N Engl J Med.

[2] Murray EJ, Hernan MA (2016). Adherence adjustment in the Coronary Drug Project: a call for better per-protocol effect estimates in randomized trials. Clin Trials.

[3] Coronary Drug Project Research Group (1973). The coronary drug project. Design, methods, and baseline results. Circulation.

[4] The Coronary Drug Project (1970). Initial findings leading to modifications of its research protocol. J Am Med Assoc.

[5] The Coronary Drug Project (1972). Findings leading to further modifications of its protocol with respect to dextrothyroxine. The Coronary Drug Project Research Group. J Am Med Assoc.

[6] The Coronary Drug Project (1973). Findings leading to discontinuation of the 2.5-mg day estrogen group. The Coronary Drug Project Research Group. JAMA.

[7] Coronary Drug Project Research Group (1975). Clofibrate and niacin in coronary heart disease. JAMA.

[8] Toh S, Hernandez-Diaz S, Logan R (2010). Estimating absolute risks in the presence of nonadherence: an application to a follow-up study with baseline randomization. Epidemiology.

[9] Cain LE, Cole SR (2009). Inverse probability-of-censoring weights for the correction of time-varying noncompliance in the effect of randomized highly active antiretroviral therapy on incident AIDS or death. Stat Med.

[10] Thompson WA (1977). On the treatment of grouped observations in life studies. Biometrics.

[11] Robins JM, Finkelstein DM (2000). Correcting for noncompliance and dependent censoring in an AIDS Clinical Trial with inverse probability of censoring weighted (IPCW) log-rank tests. Biometrics.

[12] Danaei G, Rodriguez LAG, Cantero OF, et al. Electronic medical records can be used to emulate target trials of sustained treatment strategies. J Clin Epidemiol. 2017; in press 10.1016/j.jclinepi.2017.11.021 PMC5847447 29203418

