# Supplementary material for: Improved adherence adjustment in the Coronary Drug Project
Source: Trials. 2018 Mar 5;19:158. doi: 10.1186/s13063-018-2519-5 (PMC5836455; doi:10.1186/s13063-018-2519-5)
Supplement: Supplementary file 1 — SAS 9.4 code for placebo-arm adherence analysis. A SAS code for all analyses is provided in this file. If you have any questions, comments, or discover an error, please contact Eleanor Murray at emurray@mail.harvard.edu. For the most updated versions of related SAS programs, please visit www.hsph.harvard.edu/causal/. (PDF 316 kb) [file 13063_2018_2519_MOESM1_ESM.pdf]

April 14, 2017

Improved adherence adjustment in the Coronary Drug Project

Eleanor J. Murray[1], Miguel A. Hernán[1,2,3]

1. Department of Epidemiology, Harvard T.H. Chan School of Public Health, Boston, MA
2. Department of Biostatistics, Harvard T.H. Chan School of Public Health, Boston, MA
3. Harvard-MIT Division of Health Sciences and Technology, Boston, MA

Corresponding Author: Eleanor Murray, 677 Huntington Ave, Suite 911, Boston, MA. Email: emurray@mail.harvard.edu

Trial registration number and trial register: ClinicalTrials.gov Identifier – NCT00000482

SAS Appendix

This package contains the SAS program used for the analyses in this paper. SAS 9.4 was used for all analyses. The code appendix contains the following programs.

1. Program 1: Data management
    - Takes as input cleaned CDP dataset used for our previous analyses,(1) and available through application to the National Heart, Lung, and Blood Institute.
2. Program 2: Analysis
    - Contains a macro that can produce all output in the Table, by setting macro options for &adjust (0 = unadjusted, 1 = baseline standardized, 2 = + IP weighting) and &model (see code for 6 possible values).
    - Requires the restricted cubic spline macro developed by F.E. Harrell.(2, 3)

The datasets for the Coronary Drug Project will be available through application to the National Heart, Lung, and Blood Institute. If you have any questions, comments, or discover an error, please contact Eleanor Murray at emurray@mail.harvard.edu. For the most updated versions of the SAS programs, please visit www.hsph.harvard.edu/causal/.

References

1. Murray EJ, Hernan MA. Adherence adjustment in the Coronary Drug Project: A call for better per-protocol effect estimates in randomized trials. Clin Trials. 2016;13(4):372-8.
2. Harrell FE. %rcspline macro. Clinical Biostatistics Duke University Medical Center, 1988.
3. Devlin TF, Weeks BJ, editors. Spline functions for logistic regression modeling. Proceedings of the Eleventh Annual SAS Users Group International; 1986 February 9-12; Atlanta, Georgia: Cary NC: SAS Institute.

Program 1: Data management

```
libname cdp "<path>";

data cdp.hzmodel;
set cdp.binary;

array adhFV(26)                 adhFV0 -adhFV25;
array adhbin_a(*)               adhbin0 - adhbin15;
array adhbin_aFV(*)             adhbinFV0 - adhbinFV15;
array cum_adhbin(*)             adhbin27-adhbin42;
array avgadh_t(*)               avg_adh0 - avg_adh15;
array adh(*)                    adh1-adh16;
array indic(*)                  indic0 - indic15;

array NIHA_FV_a(*)              NIHA_FV0 - NIHA_FV15;
array HiSysBP_FV_a(*)           HiSysBP_FV0-HiSysBP_FV15;
array HiDiasBP_FV_a(*)          HiDiasBP_FV0-HiDiasBP_FV15 ;
array HiWhiteCell_FV_a(*)       HiWhiteCell_FV0-HiWhiteCell_FV15;
array HiNeut_FV_a(*)            HiNeut_FV0-HiNeut_FV15;
array HiHemat_FV_a(*)           HiHemat_FV0-HiHemat_FV15;
array HiBili_FV_a(*)            HiBili_FV0-HiBili_FV15;
array HiSerChol_FV_a(*)                 HiSerChol_FV0-HiSerChol_FV15;
array HiSerTrigly_FV_a(*)       HiSerTrigly_FV0-HiSerTrigly_FV15;
array HiSerUric_FV_a(*)         HiSerUric_FV0-HiSerUric_FV15;
array HiSerAlk_FV_a(*)          HiSerAlk_FV0-HiSerAlk_FV15;
array HiPlasUrea_FV_a(*)        HiPlasUrea_FV0-HiPlasUrea_FV15;
array HiFastGluc_FV_a(*)        HiFastGluc_FV0-HiFastGluc_FV15;
array HiOneGluc_FV_a(*)         HiOneGluc_FV0-HiOneGluc_FV15;
array HiHeart_FV_a(*)           HiHeart_FV0-HiHeart_FV15;
array CHF_FV_a(*)               CHF_FV0-CHF_FV15;
array ACI_FV_a(*)               ACI_FV0-ACI_FV15;
array AP_FV_a(*)                AP_FV0-AP_FV15;
array IC_FV_a(*)                IC_FV0-IC_FV15;
array ICIA_FV_a(*)              ICIA_FV0-ICIA_FV15;
array DIG_FV_a(*)               DIG_FV0-DIG_FV15;
array DIUR_FV_a(*)              DIUR_FV0-DIUR_FV15;
array AntiArr_FV_a(*)           AntiArr_FV0-AntiArr_FV15;
array AntiHyp_FV_a(*)           AntiHyp_FV0-AntiHyp_FV15;
array OralHyp_FV_a(*)           OralHyp_FV0-OralHyp_FV15;
array CardioM_FV_a(*)           CardioM_FV0 - CardioM_FV15;
array AnyQQS_FV_a(*)            AnyQQS_FV0-AnyQQS_FV15;
array AnySTDep_FV_a(*)                  AnySTDep_FV0-AnySTDep_FV15;
array AnyTwave_FV_a(*)                  AnyTwave_FV0-AnyTwave_FV15;
array STElev_FV_a(*)            STElev_FV0-STElev_FV15;
array FVEB_FV_a(*)              FVEB_FV0-FVEB_FV15;
array VCD_FV_a(*)               VCD_FV0-VCD_FV15;
array CIG_FV_a(*)               CIG_FV0 - CIG_FV15;
array INACT_FV_a(*)             INACT_FV0-INACT_FV15;
```

```
if                      . < adhFV0 < 80 then adhbinFV0 = 1;
else if     adhFV0 >=80 then adhbinFV0 = 0;
else if     adhFV0 = .  then adhbinFV0 = .;

do i=2 to 15;
        if                      . < adhFV(i) < 80 then adhbin_aFV(i) = 1;
        else if     adhFV(i) >=80 then adhbin_aFV(i) = 0;
        else if     adhFV(i) = .  then adhbin_aFV(i) = .;
end;

avg_adh0 = adhbinFV0;

do i = 2 to 15;
        avgadh_t(i)=(avgadh_t(i-1)*(i-1)+adhbin_aFV(i))/i;
end;

do i = 1 to 15;

        ind = indic(i);

        visit = i-1; /*visit number: 0 = baseline, 1 = FV1, etc*/

        if i < 4 then do;
                if invdth = 0 then do;
                        cens = -1;
                        p_cens0 = 1;

                        adhr_t = adhbin_a(i);
                        adhr_tc = adhbin_aFV(i);
                        cum_adhr_t = cum_adhbin(i);

                        avg_adh_t = avgadh_t(i);

                        death = 0;

                        if i ge 2 then do; adhr_t1 = adhbin_a(i-1); adhr_tc1 = adhbin_aFV(i-1);end;
                        if i ge 3 then do; adhr_t2 = adhbin_a(i-2); adhr_tc2 = adhbin_aFV(i-2);end;

                        if i ge 2 then cum_adhr_t1 = cum_adhbin(i-1);
                        if i ge 3 then cum_adhr_t2 = cum_adhbin(i-2);

                        if i ge 2 then avg_adh_t1 = avgadh_t(i-1);
                        if i ge 3 then avg_adh_t2 = avgadh_t(i-2);
                end;

                else if i > invdth-1 then do;
                        cens = .;
```

```
                    p_cens0 = .;
                    adhr_t = .;
                    cum_adhr_t = .;
                    death = .;
            end;
            else if i le invdth - 1 then do;
                    cens = -1;
                    p_cens0 = 1;

                    adhr_t = adhbin_a(i);
                    adhr_tc = adhbin_aFV(i);

                    cum_adhr_t = cum_adhbin(i);
                    avg_adh_t = avgadh_t(i);

                    if invdth - 1 = i then death = 1;
                    else if  invdth - 1 > i then death = 0;

                    if death in (0,1) then do;
                            if i ge 2 then do; adhr_t1 = adhbin_a(i-1); adhr_tc1 = adhbin_aFV(i-
1);end;
                            if i ge 3 then do; adhr_t2 = adhbin_a(i-2); adhr_tc2 = adhbin_aFV(i-
2);end;
                            if i ge 2 then cum_adhr_t1 = cum_adhbin(i-1);
                            if i ge 3 then cum_adhr_t2 = cum_adhbin(i-2);
                            if i ge 2 then avg_adh_t1 = avgadh_t(i-1);
                            if i ge 3 then avg_adh_t2 = avgadh_t(i-2);
                    end;
            end;
    end;

    else if i ge 4 then do;
            if indic(i) = 0      then do;
                    cens = -1;
                    p_cens0 = 1;

                    if invdth - 1 = i then death = 1;
                    else if invdth = 0 or invdth - 1 > i then death = 0;
            end;
            else if indic(i) = 1            then do;
                    if adh(i) = .      then cens = 1;
                    else if adh(i) ne . then cens = 0;
                    p_cens0 = .;
            end;
            else if indic(i) = .            then do;
                    cens = .;
                    p_cens0 = .;
                    death = .;
```

```
end;

/*current and baseline adherence*/
if death in (0,1) then do;
	adhr_t = adhbin_a(i);
	adhr_tc = adhbin_aFV(i);

	adhr_t1 = adhbin_a(i-1);
	adhr_t2 = adhbin_a(i-2);
	adhr_t3 = adhbin_a(i-3);
	adhr_tc1 = adhbin_aFV(i-1);
	adhr_tc2 = adhbin_aFV(i-2);
	adhr_tc3 = adhbin_aFV(i-3);

	cum_adhr_t  = cum_adhbin(i);

	cum_adhr_t1 = cum_adhbin(i-1);
	cum_adhr_t2 = cum_adhbin(i-2);
	cum_adhr_t3 = cum_adhbin(i-3);

	avg_adh_t = avgadh_t(i);

	avg_adh_t1 = avgadh_t(i-1);
	avg_adh_t2 = avgadh_t(i-2);
	avg_adh_t3 = avgadh_t(i-3);

end;
else if death = . then do;
	adhr_t = .;
	adhr_tc = .;

	adhr_t1 = .;
	adhr_t2 = .;
	adhr_t3 = .;

	adhr_tc1 = .;
	adhr_tc2 = .;
	adhr_tc3 = .;

	cum_adhr_t = .;

	cum_adhr_t1 = .;
	cum_adhr_t2 = .;
	cum_adhr_t3 = .;

	avg_adh_t = .;
	avg_adh_t1 = .;
	avg_adh_t2 = .;
```

```
                    avg_adh_t3 = .;
          end;
end;

/*if death = 1 and i > 1 then adhr_t = adhbin_a(i-1);*/

if death in (0,1) then do;
          if adhr_t = . then adh_measure = 0;
          else if adhr_t ne . then adh_measure = 1;
end;
else if death = . then adh_measure = .;

/*Save covariates for visit i*/
NIHA_bin0 = NIHA_FV0;
NIHAFV = NIHA_FV_a(i);
if i > 1 then NIHAFV_t1 = NIHA_FV_a(i-1);           else NIHAFV_t1 = NIHA_bin0;
          if NIHAFV_t1 = . then NIHAFV_t1 = NIHA_FV_a(i-2);
          if NIHAFV_t1 = . then NIHAFV_t1 = NIHA_FV_a(i-3);

CHF0 = CHF_FV0;
ACI0 = ACI_FV0;
AP0 = AP_FV0;
IC0 = IC_FV0;
ICIA0 = ICIA_FV0;

CHFFV = CHF_FV_a(i);
ACIFV = ACI_FV_a(i);
APFV = AP_FV_a(i);
ICFV = IC_FV_a(i);
ICIAFV = ICIA_FV_a(i);

if i > 1 then CHFFV_t1 = CHF_FV_a(i-1);   else CHFFV_t1 = CHF0;
if i > 1 then ACIFV_t1 = ACI_FV_a(i-1);                 else ACIFV_t1 = ACI0;
if i > 1 then APFV_t1 = AP_FV_a(i-1);                   else APFV_t1 = AP0;
if i > 1 then ICFV_t1 = IC_FV_a(i-1);                   else ICFV_t1 = IC0;
if i > 1 then ICIAFV_t1 = ICIA_FV_a(i-1);   else ICIAFV_t1 = ICIA0;

          if CHFFV_t1 = . then CHFFV_t1 = CHF_FV_a(i-2);
          if ACIFV_t1 = . then ACIFV_t1 = ACI_FV_a(i-2);
          if APFV_t1  = . then APFV_t1 = AP_FV_a(i-2);
          if ICFV_t1  = . then ICFV_t1 = IC_FV_a(i-2);
          if ICIAFV_t1 = . then ICIAFV_t1 = ICIA_FV_a(i-2);

DIG0 = DIG_FV0;
DIUR0 = DIUR_FV0;
AntiArr0 = AntiARR_FV0;
AntiHyp0 = AntiHyp_FV0;
OralHyp0 = OralHyp_FV0;
```

```
DIGFV = DIG_FV_a(i);
DIURFV = DIUR_FV_a(i);
AntiArrFV = AntiARR_FV_a(i);
AntiHypFV = AntiHyp_FV_a(i);
OralHypFV = OralHyp_FV_a(i);

if i > 1 then DIGFV_t1 = DIG_FV_a(i-1);                 else DigFV_t1 = DIG0;
if i > 1 then DIURFV_t1 = DIUR_FV_a(i-1);               else DiurFV_t1 = DIUR0;
if i > 1 then AntiArrFV_t1 = AntiARR_FV_a(i-1);         else AntiArrFV_t1 = AntiArr0;
if i > 1 then AntiHypFV_t1 = AntiHyp_FV_a(i-1);         else AntiHypFV_t1 = AntiHyp0;
if i > 1 then OralHypFV_t1 = OralHyp_FV_a(i-1);         else OralHypFV_t1 = OralHyp0;

        if DIGFV_t1 = . then DIGFV_t1 = DIG_FV_a(i-2);
        if DIURFV_t1 = . then DIURFV_t1 = DIUR_FV_a(i-2);
        if AntiArrFV_t1  = . then AntiArrFV_t1 = AntiARR_FV_a(i-2);
        if AntiHypFV_t1  = . then AntiHypFV_t1 = AntiHyp_FV_a(i-2);
        if OralHypFV_t1 = . then OralHypFV_t1 = OralHyp_FV_a(i-2);

CardioM0 = CardioM_FV0;
CardioMFV = CardioM_FV_a(i);
if i > 1 then CardioMFV_t1 = CardioM_FV_a(i-1);         else CardioMFV_t1 = CardioM0;
        if CardioMFV_t1 = . then CardioMFV_t1 = CardioM_FV_a(i-2);

AnyQQS0 = AnyQQS_FV0;
AnySTDep0 = AnySTDep_FV0;
AnyTWave0 = AnyTWave_FV0;
STElev0 = STElev_FV0;
FVEB0 = FVEB_FV0;
VCD0 = VCD_FV0;
HiHeart0 = HiHeart_FV0;

AnyQQSFV = AnyQQS_FV_a(i);
AnySTDepFV = AnySTDep_FV_a(i);
AnyTWaveFV = AnyTWave_FV_a(i);
STElevFV = STElev_FV_a(i);
FVEBFV = FVEB_FV_a(i);
VCDFV = VCD_FV_a(i);
HiHeartFV = HiHeart_FV_a(i);

if i > 1 then AnyQQSFV_t1 = AnyQQS_FV_a(i-1);           else AnyQQSFV_t1 = AnyQQS0;
if i > 1 then AnySTDepFV_t1 = AnySTDep_FV_a(i-1);       else AnyStDepFV_t1 = AnyStDep0;
if i > 1 then AnyTWaveFV_t1 = AnyTWave_FV_a(i-1);       else AnyTWaveFV_t1 = AnyTWave0;
if i > 1 then STElevFV_t1 = STElev_FV_a(i-1);           else StElevFV_t1 = StElev0;
if i > 1 then FVEBFV_t1 = FVEB_FV_a(i-1);               else FVEBFV_t1 = FVEB0;
if i > 1 then VCDFV_t1 = VCD_FV_a(i-1);                 else VCDFV_t1 = VCD0;
if i > 1 then HiHeartFV_t1 = HiHeart_FV_a(i-1);         else HiHeartFV_t1 = HiHeart0;
```

```
            if AnyQQSFV_t1 = . then AnyQQSFV_t1 = AnyQQS_FV_a(i-2);
            if AnySTDepFV_t1 = . then AnySTDepFV_t1 = AnySTDep_FV_a(i-2);
            if AnyTWaveFV_t1 = . then AnyTWaveFV_t1 = AnyTWave_FV_a(i-2);
            if STElevFV_t1   = . then STElevFV_t1 = STElev_FV_a(i-2);
            if FVEBFV_t1     = . then  FVEBFV_t1 = FVEB_FV_a(i-2);
            if VCDFV_t1      = . then  VCDFV_t1 = VCD_FV_a(i-2);
            if HiHeartFV_t1 = . then  HiHeartFV_t1 = HiHeart_FV_a(i-2);

HiBili0 = HiBili_FV0;
HiSerChol0 = HiSerChol_FV0;
HiSerTrigly0 = HiSerTrigly_FV0;
HiSerUric0 = HiSerUric_FV0;
HiSerAlk0 = HiSerAlk_FV0;
HiPlasUrea0 = HiPlasUrea_FV0;
HiFastGluc0 = HiFastGluc_FV0;
HiOneGluc0 = HiOneGluc_FV0;

HiBiliFV = HiBili_FV_a(i);
HiSerCholFV = HiSerChol_FV_a(i);
HiSerTriglyFV = HiSerTrigly_FV_a(i);
HiSerUricFV = HiSerUric_FV_a(i);
HiSerAlkFV = HiSerAlk_FV_a(i);
HiPlasUreaFV = HiPlasUrea_FV_a(i);
HiFastGlucFV = HiFastGluc_FV_a(i);
HiOneGlucFV = HiOneGluc_FV_a(i);

if i > 1 then HiBiliFV_t1 = HiBili_FV_a(i-1);             else HiBiliFV_t1 = HiBili0;
if i > 1 then HiSerCholFV_t1 = HiSerChol_FV_a(i-1);       else HiSerCholFV_t1 = HiSerChol0;
if i > 1 then HiSerTriglyFV_t1 = HiSerTrigly_FV_a(i-1);   else HiSerTriglyFV_t1 = HiSerTrigly0;
if i > 1 then HiSerUricFV_t1 = HiSerUric_FV_a(i-1);       else HiSerUricFV_t1 = HiSerUric0;
if i > 1 then HiSerAlkFV_t1 = HiSerAlk_FV_a(i-1); else HiSerAlkFV_t1 = HiSerAlk0;
if i > 1 then HiPlasUreaFV_t1 = HiPlasUrea_FV_a(i-1);     else HiPlasUreaFV_t1 = HiPlasUrea0;
if i > 1 then HiFastGlucFV_t1 = HiFastGluc_FV_a(i-1);     else HiFastGlucFV_t1 = HiFastGluc0;
if i > 1 then HiOneGlucFV_t1 = HiOneGluc_FV_a(i-1);       else HiOneGlucFV_t1 = HiOneGluc0;

            if HiBiliFV_t1 = . then HiBiliFV_t1 = HiBili_FV_a(i-2);
            if HiSerCholFV_t1 = . then HiSerCholFV_t1 = HiSerChol_FV_a(i-2);
            if HiSerTriglyFV_t1 = . then HiSerTriglyFV_t1 = HiSerTrigly_FV_a(i-2);
            if HiSerUricFV_t1 = . then HiSerUricFV_t1 = HiSerUric_FV_a(i-2);
            if HiSerAlkFV_t1 = . then  HiSerAlkFV_t1 = HiSerAlk_FV_a(i-2);
            if HiPlasUreaFV_t1 = . then  HiPlasUreaFV_t1 = HiPlasUrea_FV_a(i-2);
            if HiFastGlucFV_t1 = . then  HiFastGlucFV_t1 = HiFastGluc_FV_a(i-2);
            if HiOneGlucFV_t1 = . then  HiOneGlucFV_t1 = HiOneGluc_FV_a(i-2);

HiSysBP0 = HiSysBP_FV0;
HiDiasBP0 = HiDiasBP_FV0;
```

```
HiSysBPFV = HiSysBP_FV_a(i);
HiDiasBPFV = HiDiasBP_FV_a(i);

if i > 1 then HiSysBPFV_t1 = HiSysBP_FV_a(i-1);               else HiSysBPFV_t1 = HiSysBP0;
if i > 1 then HiDiasBPFV_t1 = HiDiasBP_FV_a(i-1);             else HiDiasBPFV_t1 = HiDiasBP0;

        if HiSysBPFV_t1  = . then HiSysBPFV_t1 = HiSysBP_FV_a(i-2);
        if HiDiasBPFV_t1 = . then  HiDiasBPFV_t1 = HiDiasBP_FV_a(i-2);

CIG0 = CIG_FV0;
INACT0 = INACT_FV0;

CIGFV = CIG_FV_a(i);
INACTFV = INACT_FV_a(i);

if i > 1 then CIGFV_t1 = CIG_FV_a(i-1);    else CIGFV_t1 = CIG0;
if i > 1 then INACTFV_t1 = INACT_FV_a(i-1);         else InactFV_t1 = Inact0;

        if CIGFV_t1 = . then CIGFV_t1 = CIG_FV_a(i-2);
        if INACTFV_t1 = . then INACTFV_t1 = INACT_FV_a(i-2);

HiWhiteCell0 = HiWhiteCell_FV0;
HiNeut0         = HiNeut_FV0;
HiHemat0        = HiHemat_FV0;

HiWhiteCellFV = HiWhiteCell_FV_a(i);
HiNeutFV        = HiNeut_FV_a(i);
HiHematFV       = HiHemat_FV_a(i);

if i > 1 then HiWhiteCellFV_t1 = HiWhiteCell_FV_a(i-1); else HiWhiteCellFV_t1 = HiWhiteCell0;
if i > 1 then HiNeutFV_t1 = HiNeut_FV_a(i-1);      else HiNeutFV_t1 = HiNeut0;
if i > 1 then HiHematFV_t1 = HiHemat_FV_a(i-1);                     else HiHematFV_t1 =
HiHemat0;
        if HiWhiteCellFV_t1 = . then HiWhiteCellFV_t1 = HiWhiteCell_FV_a(i-2);
        if HiNeutFV_t1 = . then HiNeutFV_t1 = HiNeut_FV_a(i-2);
        if HiHematFV_t1 = . then HiHematFV_t1 = HiHemat_FV_a(i-2);

keep ID istat visit dth5 invdth
Adhx15Bin adhx15 itr entrydate death adhFV0 -adhFV25

adhbin0  adhr_t adhr_t1 adhr_t2 adhr_t3  adhr_tc adhr_tc1 adhr_tc2 adhr_tc3  cens p_cens0
adh_measure

cum_adhr_t cum_adhr_t1 cum_adhr_t2 cum_adhr_t3 adhbin27-adhbin42

avg_adh_t avg_adh_t1  avg_adh_t2  avg_adh_t3 adhbinFV0 - adhbinFV15  avg_adh0 -
avg_adh15
```

```
	adh1-adh16 adhbin0-adhbin15 indic0 - indic15 ind


	adhpre0bin age_bin nonwhite IRK MI_bin RBW_bin age_cat

				NIHA_bin0 HiSysBP0 HiDiasBP0 HiWhiteCell0 HiNeut0 HiHemat0
				HiBili0 HiSerChol0 HiSerTrigly0 HiSerUric0 HiSerAlk0 HiPlasUrea0
				HiFastGluc0 HiOneGluc0 HiHeart0 CHF0 ACI0 AP0
				IC0 ICIA0 DIG0 DIUR0 AntiArr0 AntiHyp0 OralHyp0
				CardioM0 AnyQQS0 AnySTDep0 AnyTWave0
				STElev0 FVEB0 VCD0 CIG0 INACT0

				NIHAFV HiSysBPFV HiDiasBPFV HiWhiteCellFV HiNeutFV HiHematFV
				HiBiliFV HiSerCholFV HiSerTriglyFV HiSerUricFV HiSerAlkFV
				HiPlasUreaFV HiFastGlucFV HiOneGlucFV HiHeartFV
				CHFFV ACIFV APFV ICFV ICIAFV DIGFV DIURFV AntiArrFV
				AntiHypFV OralHypFV CardioMFV AnyQQSFV AnySTDepFV
				AnyTWaveFV STElevFV FVEBFV VCDFV
				CIGFV INACTFV

				NIHAFV_t1 HiSysBPFV_t1 HiDiasBPFV_t1 HiWhiteCellFV_t1 HiNeutFV_t1
HiHematFV_t1
				HiBiliFV_t1 HiSerCholFV_t1 HiSerTriglyFV_t1 HiSerUricFV_t1 HiSerAlkFV_t1
				HiPlasUreaFV_t1 HiFastGlucFV_t1 HiOneGlucFV_t1 HiHeartFV_t1
				CHFFV_t1 ACIFV_t1 APFV_t1 ICFV_t1 ICIAFV_t1 DIGFV_t1 DIURFV_t1
AntiArrFV_t1
				AntiHypFV_t1 OralHypFV_t1 CardioMFV_t1 AnyQQSFV_t1 AnySTDepFV_t1
				AnyTWaveFV_t1 STElevFV_t1 FVEBFV_t1 VCDFV_t1
				CIGFV_t1 INACTFV_t1  ;
	output;
end;
run;

data cdp.hzmodel;
	set cdp.hzmodel;

	if death ne . then output;
run;
```

Program 2: Analysis

```
libname cdp "<path>";

%include 'rcspline.sas';

%macro partC(outdest = , inset = ,  titlemain = , nboot= , lib= , adjust = , mod = );
title &titlemain;
%let rawdata = &lib..&inset;
%put &rawdata;

/*Set up dataset for bootstraps and calculate restricted cubic spline of time*/
proc sort data=&rawdata out=onesample;
by id visit;
run;

data onesample ;
  set onesample end = _end_  ;
  by ID;
 retain _id ;
  if _n_ = 1 then _id = 0;
  if first.id then do;
            _id = _id + 1 ;
  end;
  if _end_ then do ;
     call symput("nids",trim(left(_id)));
  end;
/*Spline of time*/
%rcspline(visit,0,5,10,15);
run;

data ids ;
   do bsample = 1 to &nboot;
       do _id = 1 to &nids ;
           output ;
       end;
   end;
run;
proc surveyselect data= ids
         method = urs
         n= &nids
         seed = 1232
         out = _idsamples (keep = bsample _id  numberhits  )
         outall  noprint  ;
      strata bsample ;
run;
/*model 6 & 7 censor individuals who switch adherence level after baseline*/
/*create new dataset with censoring variable if model = 6 or 7*/
```

```
%if  &mod = 6 or &mod = 7%then  %do;
        data onesample;
                set onesample;

                by id;
                retain cens_new;

                if first.id then cens_new = 0;
                if adhr_tc ne adhbin0 then cens_new = 1;

        run;
%end;

data onesample;
        set onesample;
        if &mod = 2 then do;
                avg_adh_sq = avg_adh_t*avg_adh_t;
        end;
        else if &mod = 3 then do;
          avg_adh_sq = avg_adh_t*avg_adh_t;
          avg_adh_tvisit= avg_adh_t*visit;
          avg_adh_tvisit1= avg_adh_t*visit1;
          avg_adh_tvisit2= avg_adh_t*visit2;
          avg_adh_sqvisit = avg_adh_sq*visit;
          avg_adh_sqvisit1 = avg_adh_sq*visit1;
          avg_adh_sqvisit2 = avg_adh_sq*visit2;
        end;
        else if &mod = 4 then do;
                avg_adh_sq1 = avg_adh_t1*avg_adh_t1;
        end;
        else if &mod = 5 then do;
                avg_adh_sq1 = avg_adh_t1*avg_adh_t1;
                adhr_tcvisit= adhr_tc*visit;
                adhr_tcvisit1= adhr_tc*visit1;
                adhr_tcvisit2= adhr_tc*visit2;
                avg_adh_t1visit= avg_adh_t1*visit;
                avg_adh_t1visit1= avg_adh_t1*visit1;
                avg_adh_t1visit2= avg_adh_t1*visit2;
                avg_adh_sq1visit = avg_adh_sq1*visit;
                avg_adh_sq1visit1 = avg_adh_sq1*visit1;
                avg_adh_sq1visit2 = avg_adh_sq1*visit2;
        end;
        else if &mod = 7 then do;
                adhbin0visit= adhbin0*visit;
        end;
run;

/*set up model inputs*/
```

```
%if &mod = 1 %then %do;
	%let adh_form =  avg_adh_t;
%end;
%else %if &mod = 2 %then %do;
	%let adh_form = avg_adh_t avg_adh_sq ;
%end;
%else %if &mod = 3 %then %do;
	%let adh_form = avg_adh_t avg_adh_sq
		avg_adh_tvisit avg_adh_tvisit1 avg_adh_tvisit2 avg_adh_sqvisit avg_adh_sqvisit1
avg_adh_sqvisit2;
%end;
%else %if &mod = 4 %then %do;
	%let adh_form =  adhr_tc avg_adh_t1 avg_adh_sq1;
%end;
%else %if &mod = 5 %then %do;
	%let adh_form = adhr_tc avg_adh_t1 avg_adh_sq1 adhr_tcvisit adhr_tcvisit1 adhr_tcvisit2
			avg_adh_t1visit avg_adh_t1visit1 avg_adh_t1visit2 avg_adh_sq1visit
avg_adh_sq1visit1 avg_adh_sq1visit2;
%end;
%else %if &mod = 6 %then %do;
	%let adh_form = adhbin0;
%end;
%else %if &mod = 7 %then %do;
	%let adh_form = adhbin0 adhbin0visit;
%end;

/*create results dataset*/
data means_all;
bsample =.;
visit = .;
run;

%do bsample = 0 %to &nboot;

	title "Bootstrap number &bsample";

	/*set up bootstrap sample*/
	proc sort data = onesample ;
		by _id;
	run;
	data bootsample;
		merge onesample _idsamples (where = (bsample = &bsample));
		by _id;
	run;
	proc sort data = bootsample  sortsize=5G ;
		by id visit ;
	run;
```

```
%if &bsample = 0 %then %do;

	proc printto print = &outdest;
	run;

	data bootsample;
		set bootsample;
		numberhits = 1;
	run;
%end;

%if &adjust = 0 %then %do;
	/*Run crude outcome model: Pr(Y_t=1|Adherence)*/
	proc logistic data = bootsample descending ;
		%if (&mod = 6 or &mod = 7) %then %do; where cens_new = 0; %end;
		ods output ParameterEstimates = PE;
		model death = visit visit1 visit2  &adh_form ;
		freq numberhits;
	run;
%end;

%else %if &adjust = 1 %then %do;
	/*Run baseline-adjusted outcome model: Pr(Y_t=1|Adherence, baseline covariates)*/
	proc logistic data = bootsample descending ;
		%if (&mod = 6 or &mod = 7) %then %do; where cens_new = 0; %end;
		ods output ParameterEstimates = PE;
		model death = visit visit1 visit2  &adh_form
			adhpre0bin age_bin nonwhite IRK MI_bin RBW_bin
			NIHA_bin0 HiSysBP0 HiDiasBP0 HiWhiteCell0 HiNeut0 HiHemat0
			HiBili0 HiSerChol0 HiSerTrigly0 HiSerUric0 HiSerAlk0 HiPlasUrea0
			HiFastGluc0 HiOneGluc0 HiHeart0 CHF0 ACI0 AP0
			IC0 ICIA0 DIG0 DIUR0 AntiArr0 AntiHyp0 OralHyp0
			CardioM0 AnyQQS0 AnySTDep0 AnyTWave0
			STElev0 FVEB0 VCD0 CIG0 INACT0  ;
		freq numberhits;
	run;
%end;

%else %if &adjust = 2 %then %do;
	/*generate IP weights*/
	%weights(datain = bootsample, dataout = trunc, boot = &bsample);

	/*Run weighted regression model*/
	/*Pr(Yt=1|Adherence, Baseline covariates)*/
	proc logistic data = trunc  descending ;
		%if (&mod = 6 or &mod = 7) %then %do; where cens_new = 0; %end;
		ods output ParameterEstimates = PE;
```

```
                    model death = visit visit1 visit2  &adh_form
                        adhpre0bin age_bin nonwhite IRK MI_bin RBW_bin
                                NIHA_bin0 HiSysBP0 HiDiasBP0 HiWhiteCell0 HiNeut0 HiHemat0
                                HiBili0 HiSerChol0 HiSerTrigly0 HiSerUric0 HiSerAlk0 HiPlasUrea0
                                HiFastGluc0 HiOneGluc0 HiHeart0 CHF0 ACI0 AP0
                                IC0 ICIA0 DIG0 DIUR0 AntiArr0 AntiHyp0 OralHyp0
                                CardioM0 AnyQQS0 AnySTDep0 AnyTWave0
                                STElev0 FVEB0 VCD0 CIG0 INACT0  ;
                    weight stabw1;
                        freq numberhits;
                run;
        %end;

        /*Using predicted probabilities from appropriate model above, generate standardized dataset
and Kaplan-Meier survival estimates*/

        proc sql noprint;
                select ESTIMATE FORMAT =16.12 INTO: IBC_ESTIMATE separated by ' ' from pe;
        quit;
        proc sql noprint;
                select variable INTO: model separated by ' ' from PE;
        quit;

        proc means sum noprint data = pe;
                var df;
                output out = nobs (drop = _type_ _freq_ where=(_stat_ ="N"));
        run;
        proc sql noprint;
                select df into:nvar separated by ' ' from nobs;
        quit;

        /*create data for three interventions: (1) natural course: &adher = 0, adher = -1; (2) 0% non-
adherent: &adher = 1, adher = 0; (3) 100% non-adherent: &adher = 2, adher = 1*/
        /*interpretation note: 0% non-adherent translates to 100% of the time adherent to at least 80%
of medication which is the reference level of interest in the analyses*/

        %do adher = 0 %to 2;
                %let name_a = rq1;
                %let name = &name_a.&adher;

                data &name (keep = s ci adher visit numberhits);
                        set bootsample;
                        where visit = 0;
                        array var{&nvar} &model;
                        array coef{&nvar} (&ibc_estimate);

                        intercept = 1;
```

```
numberhits = 1;
s=1;

adher = &adher - 1;

/*Expand dataset and calculate predicted survival and risk for natural course*/
if &adher = 0 then do;
	do visit = 0 to 14;
		%rcspline(visit,0,5,10, 15);
		xbeta = 0;

		if &mod = 2 then do;
			avg_adh_sq = avg_adh_t*avg_adh_t;
		end;
		else if &mod = 3 then do;
			avg_adh_sq = avg_adh_t*avg_adh_t;
			avg_adh_tvisit= avg_adh_t*visit;
			avg_adh_tvisit1= avg_adh_t*visit1;
			avg_adh_tvisit2= avg_adh_t*visit2;
			avg_adh_sqvisit = avg_adh_sq*visit;
			avg_adh_sqvisit1 = avg_adh_sq*visit1;
			avg_adh_sqvisit2 = avg_adh_sq*visit2;
		end;
		else if &mod = 4 then do;
			avg_adh_sq1 = avg_adh_t1*avg_adh_t1;
		end;
		else if &mod = 5 then do;
			avg_adh_sq1 = avg_adh_t1*avg_adh_t1;
			adhr_tcvisit= adhr_tc*visit;
			adhr_tcvisit1= adhr_tc*visit1;
			adhr_tcvisit2= adhr_tc*visit2;
			avg_adh_t1visit= avg_adh_t1*visit;
			avg_adh_t1visit1= avg_adh_t1*visit1;
			avg_adh_t1visit2= avg_adh_t1*visit2;
			avg_adh_sq1visit = avg_adh_sq1*visit;
			avg_adh_sq1visit1 = avg_adh_sq1*visit1;
			avg_adh_sq1visit2 = avg_adh_sq1*visit2;
		end;
		else if &mod = 7 then do;
			adhbin0visit= adhbin0*visit;
		end;
		do i = 1 to dim(var);

			xbeta = xbeta + coef[i] *var[i];

		end;
	p = 1/(1+exp(-xbeta));
		s = s*(1-p);
```

```
                ci = 1-s;
                output;
        end;
end;

/*Expand dataset and calculate predicted survival and risk for interventions*/

else if &adher in (1, 2) then do;

                avg_adh_t = &adher - 1;

                if &mod in (4,5) then do;
                        /*models 4 and 5 require current and past adherence*/
                        adhr_tc = &adher - 1;
                        avg_adh_t1 = &adher - 1;
                end;

                if &mod in (6,7) then do;
                        adhbin0 = &adher -1 ;
                end;

        do visit = 0 to 14;
                %rcspline(visit,0,5,10, 15);
                xbeta = 0;

                if &mod = 2 then do;
                        avg_adh_sq = avg_adh_t*avg_adh_t;
                end;
                else if &mod = 3 then do;
                        avg_adh_sq = avg_adh_t*avg_adh_t;
                        avg_adh_tvisit= avg_adh_t*visit;
                        avg_adh_tvisit1= avg_adh_t*visit1;
                        avg_adh_tvisit2= avg_adh_t*visit2;
                        avg_adh_sqvisit = avg_adh_sq*visit;
                        avg_adh_sqvisit1 = avg_adh_sq*visit1;
                        avg_adh_sqvisit2 = avg_adh_sq*visit2;
                end;
                else if &mod = 4 then do;
                        avg_adh_sq1 = avg_adh_t1*avg_adh_t1;
                end;
                else if &mod = 5 then do;
                        avg_adh_sq1 = avg_adh_t1*avg_adh_t1;
                        adhr_tcvisit= adhr_tc*visit;
                        adhr_tcvisit1= adhr_tc*visit1;
                        adhr_tcvisit2= adhr_tc*visit2;
                        avg_adh_t1visit= avg_adh_t1*visit;
                        avg_adh_t1visit1= avg_adh_t1*visit1;
                        avg_adh_t1visit2= avg_adh_t1*visit2;
```

```
                    avg_adh_sq1visit = avg_adh_sq1*visit;
                    avg_adh_sq1visit1 = avg_adh_sq1*visit1;
                    avg_adh_sq1visit2 = avg_adh_sq1*visit2;
                end;
                else if &mod = 7 then do;
                    adhbin0visit= adhbin0*visit;
                end;

                do i = 1 to dim(var);

                    xbeta = xbeta + coef[i] *var[i];

                end;
            p = 1/(1+exp(-xbeta));
                s = s*(1-p);
                ci = 1-s;
                output;
            end;
        end;


    run;
%end;

/*combine all three interventions*/
data rq1;
    set rq10 rq11 rq12;
    by adher;
run;

/*calculate mean cumulative incidence and survival for each visit and intervention*/
proc means data = rq1 mean noprint;
    class visit adher;
    types visit*adher;
    var ci;
    freq numberhits;
    output out = mean_1 (drop =_type_ _freq_) mean(ci) = ;
run;

data mean_1;
    set mean_1;
    label ci = "Cumulative incidence" ;
    bsample = &bsample;
run;

/*combine across bsamples*/
data means_all;
    set means_all mean_1;
```

```
        by bsample visit;
        if bsample = . then delete;
    run;

    proc datasets library = work nolist;
        delete censadh_num0  censadh_dnom0 temp pctl rq1 pe mean_1;
    run;

    proc printto ;
    run;


%end;

proc printto print = &outdest;
run;

title "Summary";

proc sort data=means_all;
by bsample visit;
run;

/*Calculate standardized 5-year risk difference and 95% confidence interval*/
/*Comparing Always adhere to less than 80% to Always adhere to at least 80% of medication (adher: 1
vs 0)*/
proc transpose data=means_all out = temp prefix = Risk_;
var ci;
id adher;
by bsample visit;
run;

data temp;
set temp;
    mean100v0 = Risk_1 - Risk_0;
    surv_0 = 1-risk_0;
    surv_1 = 1-risk_1;
run;

proc sort data = temp;
by visit;
proc univariate data = temp (where = (bsample >0)) noprint;
    by visit;
    var mean100v0 risk_0 risk_1 surv_0 surv_1;
    output out = diffpctls (drop = _type_ _freq_) pctlpre = rd_ p0_ p1_ s0_ s1_ pctlpts = 2.5, 97.5;
run;
data sample0;
    set temp (where=(bsample = 0));
```

```
            keep  mean100v0  risk_0 risk_1 surv_0 surv_1 visit;
run;

proc sort data= diffpctls;  by visit;
run;

data final;
            merge sample0 diffpctls;
            by visit;
run;

/*output final results to .rtf file*/



proc printto print = &outdest;
run;

proc print data= final label noobs ;
var visit mean100v0 rd_2_5 rd_97_5;
title &titlemain;
title2 'Risk Difference, adjustment level = &adjust';
title3 "95% Confidence Intervals using &nboot samples" ;
run;

proc print data = final label noobs;
var visit risk_0 risk_1 p0_2_5 p0_97_5 p1_2_5 p1_97_5;
title2 'Risk, adjustment level = &adjust';
title3 "95% Confidence Intervals using &nboot samples" ;
run;

proc print data = final label noobs;
var visit surv_0 surv_1 s0_2_5 s0_97_5 s1_2_5 s1_97_5;
title2 'Survival, adjustment level = &adjust';
title3 "95% Confidence Intervals using &nboot samples" ;
run;

proc printto;
run;
%let timenow2=%sysfunc(time(), time.);
%let datenow2=%sysfunc(date(), date9.);
%put Part C is complete;
%put End time is &datenow2 / &timenow2 ;
%put Program is complete;
%put ;
*/

%mend partC;
```

```
%macro weights(datain = , dataout = , boot = );


proc printto;
run;

	/*IPW for adherence at time t - point estimate*/

	/*model for adherence measured at time t*/
	/*Numerator: Pr(Adh_measured=1|A_0, Baseline covariates)*/

	proc logistic data = &datain (where =( adhbin0 ne . and adhpre0bin ne . and  adhr_t1 ne . and
visit >0 and
			age_bin ne . and  nonwhite ne . and  IRK ne . and  MI_bin ne . and RBW_bin ne . and
			NIHA_bin0 ne . and  HiSysBP0  ne . and  HiDiasBP0 ne . and   HiWhiteCell0 ne . and
HiNeut0 ne . and   HiHemat0       ne . and
			HiBili0 ne . and   HiSerChol0  ne . and  HiSerTrigly0  ne . and  HiSerUric0  ne . and
HiSerAlk0  ne . and  HiPlasUrea0  ne . and
			HiFastGluc0  ne . and  HiOneGluc0  ne . and  HiHeart0 ne . and   CHF0 ne . and   ACI0  ne .
and  AP0 ne . and
			IC0  ne . and  ICIA0 ne . and   DIG0  ne . and  DIUR0  ne . and  AntiArr0 ne . and
AntiHyp0 ne . and   OralHyp0  ne . and
			CardioM0 ne . and   AnyQQS0 ne . and   AnySTDep0  ne . and  AnyTWave0 ne . and
			STElev0 ne . and   FVEB0 ne . and   VCD0 ne . and   CIG0 ne . and   INACT0 ne . and


			NIHAFV_t1  ne . and HiSysBPFV_t1 ne . and   HiDiasBPFV_t1 ne . and   HiWhiteCellFV_t1
ne . and   HiNeutFV_t1 ne . and   HiHematFV_t1 ne . and
			HiBiliFV_t1 ne . and   HiSerCholFV_t1 ne . and   HiSerTriglyFV_t1 ne . and
HiSerUricFV_t1 ne . and   HiSerAlkFV_t1 ne . and
			HiPlasUreaFV_t1  ne . and  HiFastGlucFV_t1  ne . and  HiOneGlucFV_t1  ne . and
HiHeartFV_t1   ne . and
			CHFFV_t1 ne . and   ACIFV_t1 ne . and   APFV_t1 ne . and   ICFV_t1 ne . and
			ICIAFV_t1 ne . and   DIGFV_t1 ne . and   DIURFV_t1 ne . and   AntiArrFV_t1 ne . and
			AntiHypFV_t1 ne . and   OralHypFV_t1 ne . and   CardioMFV_t1 ne . and   AnyQQSFV_t1
ne . and   AnySTDepFV_t1 ne . and
			AnyTWaveFV_t1 ne . and   STElevFV_t1 ne . and  FVEBFV_t1 ne . and   VCDFV_t1 ne . and
			CIGFV_t1 ne . and   INACTFV_t1 ne .)) descending;

			model adh_measure = visit visit1 visit2 adhbin0 adhr_t1
				adhpre0bin age_bin nonwhite IRK MI_bin RBW_bin

				NIHA_bin0 HiSysBP0 HiDiasBP0 HiWhiteCell0 HiNeut0 HiHemat0
				HiBili0 HiSerChol0 HiSerTrigly0 HiSerUric0 HiSerAlk0 HiPlasUrea0
				HiFastGluc0 HiOneGluc0 HiHeart0 CHF0 ACI0 AP0
				IC0 ICIA0 DIG0 DIUR0 AntiArr0 AntiHyp0 OralHyp0
```

```
                    CardioM0 AnyQQS0 AnySTDep0 AnyTWave0
                    STElev0 FVEB0 VCD0 CIG0 INACT0 ;
            freq numberhits;
            output out = adhmeas_num0 (keep=id visit mesr_0a0) p = mesr_0a0;
    run;


    /*Denominator: Pr(Adh_measured=1|Baseline covariates, Time-varying covariates)*/

    proc logistic data = &datain (where =( adhbin0 ne . and adhpre0bin ne . and  adhr_t1 ne . and 
visit >0 and
            age_bin ne . and  nonwhite ne . and  IRK ne . and  MI_bin ne . and RBW_bin ne . and
            NIHA_bin0 ne . and  HiSysBP0  ne . and  HiDiasBP0 ne . and   HiWhiteCell0 ne . and 
HiNeut0 ne . and   HiHemat0       ne . and
            HiBili0 ne . and   HiSerChol0  ne . and  HiSerTrigly0  ne . and  HiSerUric0  ne . and 
HiSerAlk0  ne . and  HiPlasUrea0  ne . and
            HiFastGluc0  ne . and  HiOneGluc0  ne . and  HiHeart0 ne . and   CHF0 ne . and   ACI0  ne . 
and  AP0 ne . and
            IC0  ne . and  ICIA0 ne . and   DIG0  ne . and  DIUR0  ne . and  AntiArr0 ne . and 
AntiHyp0 ne . and   OralHyp0  ne . and
            CardioM0 ne . and   AnyQQS0 ne . and   AnySTDep0  ne . and  AnyTWave0 ne . and
            STElev0 ne . and   FVEB0 ne . and   VCD0 ne . and   CIG0 ne . and   INACT0 ne . and

            NIHAFV_t1  ne . and HiSysBPFV_t1 ne . and   HiDiasBPFV_t1 ne . and   HiWhiteCellFV_t1 
ne . and   HiNeutFV_t1 ne . and   HiHematFV_t1 ne . and
            HiBiliFV_t1 ne . and   HiSerCholFV_t1 ne . and   HiSerTriglyFV_t1 ne . and 
HiSerUricFV_t1 ne . and   HiSerAlkFV_t1 ne . and
            HiPlasUreaFV_t1  ne . and  HiFastGlucFV_t1  ne . and  HiOneGlucFV_t1  ne . and 
HiHeartFV_t1   ne . and
            CHFFV_t1 ne . and   ACIFV_t1 ne . and   APFV_t1 ne . and   ICFV_t1 ne . and
            ICIAFV_t1 ne . and   DIGFV_t1 ne . and   DIURFV_t1 ne . and   AntiArrFV_t1 ne . and
            AntiHypFV_t1 ne . and   OralHypFV_t1 ne . and   CardioMFV_t1 ne . and   AnyQQSFV_t1 
ne . and   AnySTDepFV_t1 ne . and
            AnyTWaveFV_t1 ne . and   STElevFV_t1 ne . and  FVEBFV_t1 ne . and   VCDFV_t1 ne . and
            CIGFV_t1 ne . and   INACTFV_t1 ne .)) descending ;

            model adh_measure = visit visit1 visit2 adhbin0 adhr_t1
                    adhpre0bin age_bin nonwhite IRK MI_bin RBW_bin

                    NIHA_bin0 HiSysBP0 HiDiasBP0 HiWhiteCell0 HiNeut0 HiHemat0
                    HiBili0 HiSerChol0 HiSerTrigly0 HiSerUric0 HiSerAlk0 HiPlasUrea0
                    HiFastGluc0 HiOneGluc0 HiHeart0 CHF0 ACI0 AP0
                    IC0 ICIA0 DIG0 DIUR0 AntiArr0 AntiHyp0 OralHyp0
                    CardioM0 AnyQQS0 AnySTDep0 AnyTWave0
                    STElev0 FVEB0 VCD0 CIG0 INACT0

                    NIHAFV_t1 HiSysBPFV_t1  HiDiasBPFV_t1  HiWhiteCellFV_t1  HiNeutFV_t1 
HiHematFV_t1
```

```
                    HiBiliFV_t1  HiSerCholFV_t1  HiSerTriglyFV_t1  HiSerUricFV_t1  HiSerAlkFV_t1
                    HiPlasUreaFV_t1  HiFastGlucFV_t1  HiOneGlucFV_t1  HiHeartFV_t1
                    CHFFV_t1  ACIFV_t1  APFV_t1  ICFV_t1  ICIAFV_t1  DIGFV_t1  DIURFV_t1
AntiArrFV_t1
                    AntiHypFV_t1  OralHypFV_t1  CardioMFV_t1  AnyQQSFV_t1  AnySTDepFV_t1
                    AnyTWaveFV_t1  STElevFV_t1 FVEBFV_t1  VCDFV_t1
                    CIGFV_t1  INACTFV_t1 ;
            freq numberhits;
            output out = adhmeas_dnom0 (keep=id visit mesr_wa0) p = mesr_wa0;
    run;

    /*model for adherence at time t, given adherence measured*/
    /*Numerator: Pr(A_t=1|A_0, Baseline covariates)*/

    proc logistic data= &datain (where =( adhbin0 ne . and adhr_t ne . and  adhr_t1 ne . and
adhpre0bin ne . and visit >0 and
            age_bin ne . and  nonwhite ne . and  IRK ne . and  MI_bin ne . and RBW_bin ne . and
            NIHA_bin0 ne . and  HiSysBP0  ne . and  HiDiasBP0 ne . and   HiWhiteCell0 ne . and
HiNeut0 ne . and   HiHemat0       ne . and
            HiBili0 ne . and   HiSerChol0  ne . and  HiSerTrigly0  ne . and  HiSerUric0  ne . and
HiSerAlk0  ne . and  HiPlasUrea0  ne . and
            HiFastGluc0  ne . and  HiOneGluc0  ne . and  HiHeart0 ne . and   CHF0 ne . and   ACI0  ne .
and  AP0 ne . and
            IC0  ne . and  ICIA0 ne . and   DIG0  ne . and  DIUR0  ne . and  AntiArr0 ne . and
AntiHyp0 ne . and   OralHyp0  ne . and
            CardioM0 ne . and   AnyQQS0 ne . and   AnySTDep0  ne . and  AnyTWave0 ne . and
            STElev0 ne . and   FVEB0 ne . and   VCD0 ne . and   CIG0 ne . and   INACT0 ne . and

            NIHAFV ne . and   HiSysBPFV ne . and   HiDiasBPFV ne . and   HiWhiteCellFV ne . and
HiNeutFV ne . and   HiHematFV ne . and
            HiBiliFV  ne . and  HiSerCholFV ne . and   HiSerTriglyFV ne . and   HiSerUricFV ne . and
HiSerAlkFV ne . and
            HiPlasUreaFV ne . and   HiFastGlucFV ne . and   HiOneGlucFV  ne . and  HiHeartFV  ne .
and
            CHFFV  ne . and  ACIFV  ne . and  APFV  ne . and  ICFV  ne . and  ICIAFV  ne . and  DIGFV
ne . and  DIURFV ne . and   AntiArrFV  ne . and
            AntiHypFV ne . and   OralHypFV  ne . and  CardioMFV  ne . and  AnyQQSFV ne . and
AnySTDepFV ne . and
            AnyTWaveFV  ne . and  STElevFV ne . and   FVEBFV ne . and   VCDFV ne . and
            CIGFV ne . and   INACTFV ne .)) descending;

            model adhr_t = visit visit1 visit2 adhbin0 adhr_t1
                    adhpre0bin age_bin nonwhite IRK MI_bin RBW_bin

                    NIHA_bin0 HiSysBP0 HiDiasBP0 HiWhiteCell0 HiNeut0 HiHemat0
                    HiBili0 HiSerChol0 HiSerTrigly0 HiSerUric0 HiSerAlk0 HiPlasUrea0
                    HiFastGluc0 HiOneGluc0 HiHeart0 CHF0 ACI0 AP0
                    IC0 ICIA0 DIG0 DIUR0 AntiArr0 AntiHyp0 OralHyp0
```

```
			CardioM0 AnyQQS0 AnySTDep0 AnyTWave0
			STElev0 FVEB0 VCD0 CIG0 INACT0 ;
		freq numberhits;
		output out = censadh_num0 (keep=id visit punc_0a0) p = punc_0a0;
		run;

	/*Denominator: Pr(A_t=1|A_0, Baseline covariates, Time-varying covariates)*/

	proc logistic data=&datain (where =( adhbin0 ne . and adhr_t ne .  and  adhr_t1 ne .  and
adhpre0bin ne . and  visit >0 and
		age_bin ne . and  nonwhite ne . and  IRK ne . and  MI_bin ne . and RBW_bin ne . and
		NIHA_bin0 ne . and  HiSysBP0  ne . and  HiDiasBP0 ne . and   HiWhiteCell0 ne . and
HiNeut0 ne . and   HiHemat0       ne . and
		HiBili0 ne . and   HiSerChol0  ne . and  HiSerTrigly0  ne . and  HiSerUric0  ne . and
HiSerAlk0  ne . and  HiPlasUrea0  ne . and
		HiFastGluc0  ne . and  HiOneGluc0  ne . and  HiHeart0 ne . and   CHF0 ne . and   ACI0  ne .
and  AP0 ne . and
		IC0  ne . and  ICIA0 ne . and   DIG0  ne . and  DIUR0  ne . and  AntiArr0 ne . and
AntiHyp0 ne . and   OralHyp0  ne . and
		CardioM0 ne . and   AnyQQS0 ne . and   AnySTDep0  ne . and  AnyTWave0 ne . and
		STElev0 ne . and   FVEB0 ne . and   VCD0 ne . and   CIG0 ne . and   INACT0 ne . and

		NIHAFV ne . and   HiSysBPFV ne . and   HiDiasBPFV ne . and   HiWhiteCellFV ne . and
HiNeutFV ne . and   HiHematFV ne . and
		HiBiliFV  ne . and  HiSerCholFV ne . and   HiSerTriglyFV ne . and   HiSerUricFV ne . and
HiSerAlkFV ne . and
		HiPlasUreaFV ne . and   HiFastGlucFV ne . and   HiOneGlucFV  ne . and  HiHeartFV  ne .
and
		CHFFV  ne . and  ACIFV  ne . and  APFV  ne . and  ICFV  ne . and  ICIAFV  ne . and  DIGFV
ne . and  DIURFV ne . and   AntiArrFV  ne . and
		AntiHypFV ne . and   OralHypFV  ne . and  CardioMFV  ne . and  AnyQQSFV ne . and
AnySTDepFV ne . and
		AnyTWaveFV  ne . and  STElevFV ne . and   FVEBFV ne . and   VCDFV ne . and
		CIGFV ne . and   INACTFV ne .)) descending;

		model adhr_t =  visit visit1 visit2 adhbin0 adhr_t1
			adhpre0bin age_bin nonwhite IRK MI_bin RBW_bin

			NIHA_bin0 HiSysBP0 HiDiasBP0 HiWhiteCell0 HiNeut0 HiHemat0
			HiBili0 HiSerChol0 HiSerTrigly0 HiSerUric0 HiSerAlk0 HiPlasUrea0
			HiFastGluc0 HiOneGluc0 HiHeart0 CHF0 ACI0 AP0
			IC0 ICIA0 DIG0 DIUR0 AntiArr0 AntiHyp0 OralHyp0
			CardioM0 AnyQQS0 AnySTDep0 AnyTWave0
			STElev0 FVEB0 VCD0 CIG0 INACT0

			NIHAFV HiSysBPFV HiDiasBPFV HiWhiteCellFV HiNeutFV HiHematFV
			HiBiliFV HiSerCholFV HiSerTriglyFV HiSerUricFV HiSerAlkFV
			HiPlasUreaFV HiFastGlucFV HiOneGlucFV HiHeartFV
```

```
                    CHFFV ACIFV APFV ICFV ICIAFV DIGFV DIURFV AntiArrFV
                    AntiHypFV OralHypFV CardioMFV AnyQQSFV AnySTDepFV
                    AnyTWaveFV STElevFV FVEBFV VCDFV
                    CIGFV INACTFV;
        freq numberhits;
        output out = censadh_dnom0 (keep = id  visit punc_wa0)  p = punc_wa0;
run;


proc sort data=adhmeas_num0;
        by ID visit;
proc sort data=adhmeas_dnom0;
        by ID visit;
proc sort data=censadh_num0;
        by ID  visit;
proc sort data=censadh_dnom0;
        by ID  visit;
proc sort data=&datain;
        by ID  visit;
data main_w6;
        merge &datain censadh_num0 censadh_dnom0 adhmeas_num0 adhmeas_dnom0 ;
        by ID  visit;

        /* variables ending with _0 refer to the numerator of the weights
          Variables ending with _w refer to the denominator of the weights */

     if first.id then do;
                    k1_0=1;
                    k1_w=1;
                    m1_0=1;
                    m1_w=1;
        end;
        retain k1_0 k1_w m1_0 m1_w;

        if adhr_t ne . then do;

                    if mesr_0a0 = . then mesr_0a0 =1;
                    if mesr_wa0 = . then mesr_wa0 =1;
                    m1_0=m1_0*mesr_0a0;
                    m1_w=m1_w*mesr_wa0;

                    if adhr_t = 0 then do;
                            if punc_0a0 = . then punc_0a0 =0;
                            if punc_wa0 = . then punc_wa0 =0;

                        k1_0=k1_0*(1-punc_0a0);
                            k1_w=k1_w*(1-punc_wa0);
                    end;
```

```
                else if adhr_t = 1 then do;
                        if punc_0a0 = . then punc_0a0 =1;
                        if punc_wa0 = . then punc_wa0 =1;
                    k1_0=k1_0*(punc_0a0);
                k1_w=k1_w*(punc_wa0);
                end;

        end;
        else if adhr_t = . then do;
                if mesr_0a0 = . then mesr_0a0 =0;
                if mesr_wa0 = . then mesr_wa0 =0;
                m1_0=m1_0*(1-mesr_0a0);
                m1_w=m1_w*(1-mesr_wa0);

                k1_0 = k1_0*1;
                k1_w = k1_w*1;
        end;

    stabw_a=(k1_0)/(k1_w);
    nstabw_a=1/(k1_w);

    stabw_m=(m1_0)/(m1_w);
    nstabw_m=1/(m1_w);

    stabw =stabw_a*stabw_m;
    nstabw =nstabw_a*nstabw_m;

run;

%if &boot = 0 %then %do;
        proc printto print = &outdest;
        run;
        proc means data=main_w6  n mean std min max p95 p99 nmiss;
                var nstabw stabw nstabw_a stabw_a nstabw_m stabw_m;
                title 'weights, all';
        run;
        proc printto print = &outdest;
        run;
        proc freq data=main_w6 nlevels;
                where stabw ne .;
                tables ID /noprint;
                title 'weights, all';
        run;
%end;

/*for truncation*/
proc means data=main_w6  n mean std min max p95 p99 nmiss noprint;
        var stabw;
```

```
		title 'stabilized weights, end of follow-up';
		output out=pctl (keep = p99) p99 = p99 ;
	run;
	proc means data=main_w6  p99 noprint;
		var nstabw;
		title 'stabilized weights, end of follow-up';
		output out=pctl_n (keep = p99) p99 = p99 ;
	run;

	data temp;
		set pctl;
		call symput ('cutoff', p99);
	run;
	data temp_n;
		set pctl_n;
		call symput ('cutoff_n', p99);
	run;

	data &dataout;
		set main_w6;
		stabw1 = stabw;
		if stabw >  %sysevalf(&cutoff)  then do;
			stabw1 = %sysevalf(&cutoff);
		end;
		nstabw1 = nstabw;
		if nstabw >  %sysevalf(&cutoff_n)  then do;
			nstabw1 = %sysevalf(&cutoff_n);
		end;
	run;

	%if &boot = 0 %then %do;

		proc printto print = &outdest;
		run;

		proc means data=&dataout  n mean std min max p95 p99 nmiss;
			var stabw stabw1 nstabw nstabw1;
			title 'stabilized weights, end of follow-up';
		run;
	%end;

%mend;


/**RUN MACROS**/

%let nboot = 5;
```

```
%partC( outdest = "output.rtf", inset = hzmodel, titlemain = 'Adherence at time t', nboot = &nboot,
lib=cdp, adjust = 0, mod = 1);

****&adjust specifies level of adjustment: 0 = unadjusted; 1 = baseline adjusted; 2 = post-baseline
adjusted
****&mod specifies model for adherence: see below
/*model1 - linear:
			model death = visit visit1 visit2 avg_adh_t*/
/*model2 - quadratic:
			model death = visit visit1 visit2 avg_adh_t avg_adh_sq */
/*model3 - quadratic with time interaction:
			model death = visit visit1 visit2 avg_adh_t avg_adh_sq
			avg_adh_tvisit avg_adh_tvisit1 avg_adh_tvisit2 avg_adh_sqvisit avg_adh_sqvisit1
avg_adh_sqvisit2*/
/*model4 - binary current adherence plus quadratic cumulative average to previous visit:
			model death = visit visit1 visit2 adhr_tc avg_adh_t1 avg_adh_sq1*/
/*model5 - binary current adherence plus quadratic cumulative average to previous visit, with time
interaction:
			model death = visit visit1 visit2 adhr_tc avg_adh_t1 avg_adh_sq1 adhr_tcvisit
adhr_tcvisit1 adhr_tcvisit2
				avg_adh_t1visit avg_adh_t1visit1 avg_adh_t1visit2 avg_adh_sq1visit
avg_adh_sq1visit1 avg_adh_sq1visit2*/
/*model6 - binary baseline adherence with censoring if adherence deviates from baseline:
			model death = visit visit1 visit2 adhbin0*/
/*model7 - binary baseline adherence with censoring if adherence deviates from baseline and time
interaction:
			model death = visit visit1 visit2 adhbin0 adhbin0visit */
```
